# Summary of the National Advisory Committee on Immunization (NACI) Rapid Response: Updated interim guidance on Imvamune in the context of ongoing monkeypox outbreaks

**DOI:** 10.14745/ccdr.v48i1112a11

**Published:** 2022-11-03

**Authors:** Nicole Forbes, Oliver Baclic, Robyn Harrison, Nicholas Brousseau

**Affiliations:** 1 Public Health Agency of Canada, Centre for Immunization and Respiratory Infectious Diseases, Ottawa, ON; 2 Alberta Health Services, Workplace Health and Safety Program, Edmonton, AB; 3 Institut national de santé publique du Québec, Direction des risques biologiques, Québec, QC

**Keywords:** National Advisory Committee on Immunization, monkeypox, Canada, Imvamune, outbreak guidance

## Abstract

**Background:**

During the period of monkeypox community transmission and restricted vaccine supply in the summer of 2022, Canadian provinces and territories and a number of vaccine stakeholders indicated the need for consistent national guidance on pre-exposure vaccination (including the identification of priority populations for pre-exposure vaccination programs) and guidance on the potential use of dose-sparing strategies.

**Methods:**

The National Advisory Committee on Immunization (NACI) High Consequence Infectious Disease Working Group reviewed data on the status of the monkeypox outbreak along with additional published and non-published evidence regarding the safety, immunogenicity and protection offered by Imvamune^®^. NACI approved updated recommendations on September 16, 2022, and on September 23, 2022 it released updated interim guidance on the use of Imvamune in the context of the ongoing monkeypox outbreak.

**Results:**

During periods of adequate vaccine supply, NACI recommended that Imvamune pre-exposure vaccination should be offered as a two-dose primary series, with at least 28 days between the two sub-cutaneous doses. When supply is limited, guidance was provided for the use of dose sparing strategies, including extended dosing intervals and fractional intradermal dosing to maximize vaccine coverage for those at highest risk of exposure to the monkeypox virus.

**Conclusion:**

The updated NACI recommendations provide additional guidance on the use of Imvamune for the management of the 2022 monkeypox outbreak in Canada and may be considered to maximize vaccine coverage in outbreak settings when supply is limited.

## Introduction

On June 10, 2022, in the context of a rapidly evolving monkeypox outbreak, National Advisory Committee on Immunization (NACI) provided options for the use of the Imvamune^®^ vaccine (Modified vaccinia Ankara Bavarian Nordic; MVA-BN) for post-exposure vaccination against monkeypox ((1)). NACI recommended that a single dose of the Imvamune vaccine may be offered to people with high-risk exposures to a probable or confirmed case of monkeypox, or within a setting where transmission is happening; a second dose could be offered after 28 days only if an assessment indicated an ongoing risk of exposure ((1)).

Canadian jurisdictions experiencing ongoing monkeypox outbreaks built on the foundation of the early NACI guidance on the use of Imvamune. Specifically, jurisdictions with active monkeypox outbreaks expanded eligibility for Imvamune vaccine administration beyond post-exposure use based in part on the limited feasibility of case and contact identification with this outbreak.

The Public Health Agency of Canada (PHAC), together with the provinces and territories, identified the need for national guidance on pre-exposure vaccination, including identification of priority populations for pre-exposure vaccination programs and guidance on the potential use of dose-sparing strategies (i.e. extended dosing intervals and/or fractional intradermal dosing).

The 2022 monkeypox outbreaks in Canada, the United States and Europe have primarily affected men who identify as men who have sex with men (MSM) and who have reported recent sex with one or multiple partners ((2)). The majority of cases reported no contact with a person known to have a confirmed monkeypox infection ((3–5)). The severity of disease reported in the 2022 Canadian outbreaks has been generally low, with fewer reported hospitalizations, intensive care unit (ICU) admissions and deaths (case fatality rate of less than 0.1%) compared with historical outbreaks ((5–8)). At least 25% of cases were reported to have a concomitant sexually transmitted infection ((3,7,9–11)).

For the purposes of the NACI Statement, MSM is defined as: man or Two-Spirit identifying individual who has sex with another person who identifies as a man, including but not limited to individuals who self-identify as transgender, cis-gender, Two-Spirit, gender-queer, intersex and non-binary and who also identify as gay, bisexual or pansexual.

## Methods

On August 22, 2022, the NACI High Consequence Infectious Disease Working Group (HCID WG) was convened to discuss and review data on the evolving monkeypox outbreak. Input was sought from and provided by the Public Health Ethics Consultative Group, Canadian Immunization Committee, NACI’s Vaccine Safety Working Group and the National Emergency Strategic Stockpile. That same date, Montréal Public Health and Ontario Ministry of Health presented emerging evidence on the ongoing monkeypox outbreaks, including epidemiological trends and Imvamune vaccine programs to the HCID WG. Three groups representing 2SLGBTQI+ communities and one group representing sex workers were consulted to provide stakeholder input on the acceptability of vaccine strategies.

The HCID WG reviewed data on the current status of the monkeypox outbreak in Canada and globally, along with additional evidence included in published scientific literature and from the manufacturer, regarding the safety, immunogenicity and protection offered by Imvamune. Modelling information provided by PHAC on the impact of dose sparing strategies when vaccine supply is limited was also reviewed.

## Results

By September 16, 2022, nine Canadian provinces and territories had publicly reported 1,363 cases of monkeypox ((3)). Over 95% of confirmed cases have been in men 18–44 years of age who self-identified as gay, bisexual and other MSM and as having multiple and/or new sex partners; 52% reported living with human immunodeficiency virus (HIV). In response to the outbreak, PHAC had distributed over 110,000 doses of Imvamune vaccine to provinces and territories, and over 70,000 people had been vaccinated with at least one dose as of August 28, 2022 ((12)). The epidemiology of Canadian and international outbreaks has helped identify individuals and groups at highest risk of exposure to the virus. Men who have sex with men and individuals who have sex with MSM have the highest risk of being exposed to the monkeypox virus, provided they have multiple sex partners, have had a recent sexually transmitted infection or engage in sexual contact at sex-on-premise venues. Individuals who self-identify as sex worker, regardless of self-identified sex or gender, sand individuals who volunteer or work at sex-on-premise venues may also be at higher risk of exposure to the monkeypox virus.

Available post-marketing data on Imvamune safety collected until September 2022 provided assurances that the vaccine was well tolerated when administered prophylactically ((13,14)). In Canada, the majority of adverse events following immunization reported to the passive surveillance system were non-serious and primarily include injection site reactions and fatigue (*personal communication, Public Health Agency of Canada; Surveillance of adverse events following immunization with Imvamune. August 17, 2022*).

The HCID WG did not identify any direct evidence on the efficacy or effectiveness of a two-dose primary series of Imvamune (given as either pre or post-exposure vaccination) against monkeypox infection, transmission or severe disease. Emerging evidence suggested that individuals vaccinated with one dose of Imvamune and who remained at high risk of exposure following vaccination could be at risk of infection post-vaccination ((13,15)).

Real world, experimental and modelling data provided evidence that extended two-dose intervals and intradermal vaccine administration could provide protection from monkeypox infection at an individual level while maximizing vaccine coverage for those at highest risk of monkeypox exposure ((16–19)).

A smaller intradermal (ID) dose, administered between layers of the skin, is expected to generate a similar immune response to a full dose administered subcutaneously (SC) but requires technical skill and careful planning in order to prevent vaccine dose wastage and to ensure safety given the multi-dose vial vaccine preparations with limited shelf life once opened ((16)). Intradermal administration of vaccine (for dose sparing) thus poses feasibility challenges. Broad and safe deployment of ID doses may be optimal when used for second doses but not for first doses. In addition, there is a large body of evidence regarding the on-label (SC), administration of Imvamune. Internal PHAC modelling based on Canadian supply projections suggested that expanding vaccine coverage by extending dose intervals of the Imvamune vaccine and using 1-full (SC) and 1-fractional (ID) dose could have short-term public health benefits in preventing infections while vaccine supply is constrained. This unique potential solution stems from what is known about different vaccination strategies, principles of vaccinology, and feasibility of vaccination programs.

## Recommendations

Following the review of available evidence, NACI made the following recommendations.

### Pre-exposure vaccination

1.1 In the context of an active monkeypox outbreak, NACI recommends that immunization using the Imvamune vaccine should be offered to individuals with highest risk of monkeypox. After considering current and projected outbreak epidemiology, NACI recommends the following individuals/groups be considered for vaccination with Imvamune:

MSM and individuals who have sex with MSM, and who meet at least one of the following criteria:

• Having two or more sexual partners or being in a relationship where at least one of the partners has other sexual partners

• Having had a confirmed sexually transmitted infection acquired in the last year

• Engage in sexual contact in sex-on-premise venues

OR

• Individuals who self-identify as sex workers regardless of self-identified sex/gender

OR

• Staff or volunteers in sex-on-premise venues where workers may have contact with fomites potentially contaminated with monkeypox, without the use of personal protective equipment

The NACI continues to recommend pre-exposure vaccination with Imvamune vaccine for those working in research laboratory settings with replicating orthopoxviruses as outlined in the June 10, 2022 *NACI Rapid Response, Updated interim guidance on Imvamune® in the context of ongoing monkeypox outbreaks*.

1.2. Those with prior documented history of monkeypox infection need not be vaccinated. **(Strong NACI recommendation)**

2. In the context of the ongoing monkeypox outbreak and limited vaccine supply, dose sparing strategies should be considered in order to expand vaccination coverage to a broader population currently considered for pre-exposure vaccination. **(Strong NACI recommendation)**

2.1. Among immunocompetent adults currently considered for pre-exposure vaccination, the first dose of Imvamune can be prioritized in order to extend the potential protective impact broadly across populations most at risk of exposure.

Second doses should be offered as soon as demand for first doses among eligible individuals has been met. Individuals should receive their second dose at least 28 days after the first dose, provided they are at ongoing risk of exposure. This may result in an extended interval strategy, where the second dose is offered beyond the minimum authorized interval (28 days).

Individuals considered moderately to severely immunocompromised and currently eligible for pre-exposure vaccination should be prioritized to receive two doses of the Imvamune vaccine administered at the authorized interval (28 days between doses).

2.2. NACI recommends that, in the context of limited Imvamune vaccine supply, off-label ID administration (0.1 mL per dose) can be used among immunocompetent adults when given as a second dose following a first dose given subcutaneously, provided dose sparing and safe administration practises are feasible.

Individuals who are younger than 18 years of age, at risk of keloid scars, or moderately to severely immunocompromised should be offered Imvamune vaccine using the subcutaneous route of administration only.

Personnel involved in preparing and administering the vaccine should be provided adequate training before implementing intradermal administration. Jurisdictions should have protocols to minimize the risk of dose wastage and to reduce the potential of contamination of the vials if single-dose vials are to be used for multiple doses. If a vial is used for multiple doses, it should be discarded after six hours following first puncture.

3. NACI recommends that, when supply is not constrained, Imvamune pre-exposure vaccination should be offered as a two-dose primary series, with at least 28 days between first and second SC doses, for individuals currently eligible for pre-exposure vaccination. **(Strong NACI recommendation)**

### Post-exposure vaccination

4. NACI continues to recommend the use of Imvamune as a post-exposure vaccination (also known and referred to as post-exposure prophylaxis) to individuals who have had high risk exposure(s) to a probable or confirmed case of monkeypox, or within a setting where transmission is happening. A post-exposure vaccine dose should be offered as soon as possible, preferably within four days of last exposure but can be considered up to 14 days of last exposure. It should not be offered to individuals who are symptomatic and who meet the definition of suspect, probable or confirmed case. **(Strong NACI Recommendation)**

A summary table of the recommended immunization schedule is provided in **Appendix**.

## Conclusion

The updated NACI recommendations identify groups at risk of monkeypox during the 2022 ongoing outbreak in Canada that are eligible for pre-exposure vaccination and provide additional strategies on the use of Imvamune that may be considered in order to maximize vaccine coverage when vaccine supply is limited. The future course monkeypox epidemiology remains unknown; thus, as the current outbreak evolves and new risk factors or groups at higher risk are identified, the criteria for those who should be vaccinated may change.

## Appendix Summary table (immunization schedule)

**Table A1:** Immunization schedule for Imvamune^®^ in the context of the 2022 monkeypox outbreak

Dose number	Pre-exposure vaccination^a,b^		Post-exposure vaccination^a,b^	
	Immunocompetent adults	Moderately to severely immunocompromised and/or younger than 18 years of age and/or increased risk of keloid scars	Immunocompetent adults	Moderately to severely immunocompromised and/or younger than 18 years of age and/or increased risk of keloid scars
Dose 1	0.5 mL, SC	0.5 mL, SC	0.5 mL, SC, within 4 days since exposure, can be considered up to 14 days	0.5 mL, SC within 4 days since exposure, can be considered up to 14 days
Dose 2	0.5 mL, SC, 28 days after dose 1 (supply not constrained) OR 0.5 mL SC administered ≥28 days after dose 1 (constrained supply) OR 0.1 mL, ID (constrained supply only)	0.5 mL, SC 28 days after dose 1	0.5 mL, SC (if at ongoing risk of exposure)	0.5 mL, SC (if at ongoing risk of exposure)

Abbreviations: ID, intradermal; SC, subcutaneous

^a^ Immunocompetent individuals recommended for Imvamune pre-exposure or post-exposure vaccination should receive a single dose if they have previously been vaccinated with a live replicating 1^st^ or 2^nd^ generation smallpox vaccine (i.e. as a booster dose). However, individuals considered moderately to severely immunocompromised should receive two doses, regardless of previous smallpox vaccination

^b^ Pre-exposure or post-exposure vaccination is not indicated for individuals who meet the definition of suspect, probable or confirmed monkeypox case or with prior history of infection with monkeypox

In the context of constrained supply, for immunocompetent individuals, the first dose can be prioritized; this may result in an extended interval strategy, where the second dose is offered beyond the minimum authorized interval of 28 days. For post-exposure vaccination, the second dose is only administered if the person is at ongoing risk of exposure.

Imvamune given as pre-exposure or post-exposure vaccination should not be delayed due to recent receipt of a messenger ribonucleic acid (mRNA) coronavirus disease 2019 (COVID-19) vaccine. If vaccine timing can be planned (i.e. prior to employment within a research laboratory), NACI recommends that Imvamune be given at least four weeks after or before an mRNA vaccine for COVID-19. Refer to the June 10, 2022, NACI Statement for details on co-administration guidance.
